# Pharmacists’ Perceptions of the Benefits and Challenges of Electronic Product Information System Implementation in Hong Kong: Mixed-Method Study

**DOI:** 10.2196/20765

**Published:** 2020-11-10

**Authors:** Eunice Wing To Fung, Gordon Tsz Fung Au-Yeung, Lo Mei Tsoi, Lili Qu, Tommy Kwan Wa Cheng, Donald Wing-Kit Chong, Teddy Tai Ning Lam, Yin Ting Cheung

**Affiliations:** 1 School of Pharmacy, Faculty of Medicine The Chinese University of Hong Kong Hong Kong China (Hong Kong); 2 Pfizer Corporation Hong Kong Limited Hong Kong China (Hong Kong); 3 GlaxoSmithKline Consumer Healthcare (Hong Kong) Limited Hong Kong China (Hong Kong)

**Keywords:** electronic product information, drug information system, electronic health information, health care professionals, retrieval of health information

## Abstract

**Background:**

With the advancement of technology, more countries are now adopting the use of electronic product information (ePI), which refer to an electronic version of physical product inserts in a semistructured format optimized for electronic manipulation. The successful implementation of ePI has led to advantages and convenience to patients, health care professionals, and pharmaceutical companies in many regions and countries. In the Hong Kong Special Administrative Region (SAR), there is currently no citywide implementation of ePI. The SAR exhibits conditions that would favor the implementation of an ePI system, as well as existing barriers hindering its implementation. However, no study has been performed to examine the specific situation in Hong Kong.

**Objective:**

The objective of this study is to explore working pharmacists’ overall perception of ePI and to identify potential challenges to the implementation of an ePI system in Hong Kong.

**Methods:**

This mixed-method study involved a structured survey and interview with practicing pharmacists in Hong Kong. Pharmacists were eligible if they were licensed to practice in Hong Kong, and currently working locally in any pharmacy-related sectors and institutions. Respondents completed a survey to indicate their level of agreement with statements regarding the potential advantages of ePI over paper PI. A structured interview was conducted to gather respondents’ perceived advantages of ePI over paper PI in different aspects, such as professionalism, usability, presentation, and environment, as well as challenges of citywide ePI implementation in Hong Kong. Thematic analysis was adopted to analyze the qualitative data. Grounded theory was used to generate themes and identify specific outcomes.

**Results:**

A total of 16 pharmacists were recruited, comprising 4 community pharmacists, 5 hospital pharmacists, and 7 industrial pharmacists. All of them used electronic platforms at least once per month on average. Respondents identified many flaws in physical package inserts that can potentially be mitigated using ePI. The speed with which drug information can be retrieved and the degree to which the drug information can be readily updated and disseminated were considered the greatest strengths of ePI. The clarity with which ePI present drug information to patients was considered as the weakest aspect of ePI. Many respondents highlighted concerns about the security risks and high cost associated with system maintenance and that certain subpopulations may not be sufficiently computer literate to navigate the ePI system. Respondents also voiced many concerns about the implementation and maintenance of a local ePI system.

**Conclusions:**

We conclude that an ePI system is generally supported by pharmacists but concerns about implementation process and maintenance of the system has been raised. The perceived benefits of ePI gathered from this study, as well as collective evidence from other countries with mature ePI systems, confirm that more efforts should be made to promote optimized development and implementation of an ePI system in Hong Kong.

## Introduction

Product inserts in the packaging of medicines typically include a summary of product characteristics (SmPCs) and a package leaflet, and are regulated and legally required in most countries. The accelerating progress of technology has also led to the development of electronic product information (ePI) for medicines, and this is now gaining popularity in Australia, Canada, Switzerland, Japan, the United States, and countries within the European Union (EU) [1-5].

According to an international report published by the European Medicines Agency (EMA), most regions and countries regard ePI as product information for electronic handling. It is composed of an SmPC, a package leaflet, and product labeling in a semistructured format under a common electronic standard stipulated by governing bodies [2]. Common electronic standards comprise mark-up languages, standardized vocabularies, and internationally recognized interoperability specifications. Specifically, text is annotated to formats such as eXtensible Markup Language (XML), JavaScript Object Notation (JSON), and HyperText Markup Language (HTML), with XML being the most commonly adopted format. Standardized vocabularies are used to describe medicine information, to enable searches across different product information (PI). Interoperability specifications enable the integration of ePI into other health systems to facilitate its use by a wide range of health care professionals. Its semistructured format allows the inclusion of structured elements (fixed headings and controlled vocabularies) and unstructured elements (free text and graphics) to tailor product information to individual needs [2]. Most ePI formats can be disseminated via the web, e-platforms (software or websites and tools such as computers, mobile devices, and wearables), and print media.

Different studies of health care professionals’ perceptions of ePI have been conducted in various countries. Ahead of the launch of an ePI pilot scheme in Belgium and Luxembourg, results of a survey of hospital pharmacists demonstrated strong support for the use of ePI for patient services [6]. Ninety percent of the respondents supported a complete switch from paper to electronic versions of product information, and up to 55% of the respondents used only electronic package “leaflets” in their daily practice. Another study examined pharmacists’ readiness for paperless labeling in the United States [7], and a study assessing community pharmacists’ perception of tailor-made electronic leaflets was also conducted in Portugal in 2018 [8]. The results of both studies showed that pharmacists had positive attitudes about the use of individually tailored patient leaflets to address the individual needs of their patients, and many pharmacists believed that such initiatives could improve the health literacy of patients. In general, the results of studies performed in many countries have demonstrated that pharmacists strongly support the implementation of ePI systems.

In the Hong Kong Special Administrative Region (SAR), there is currently no citywide implementation of ePI. However, the SAR exhibits several conditions that would favor the implementation of an ePI system. For example, there is a clear set of laws in place to regulate the registration and labeling of pharmaceutical products in Hong Kong [9]. In addition, there is a high penetration rate of technology in Hong Kong: in 2018, over 90% of households had access to the internet at home [10]. Moreover, more than 90% of people aged 10 years or over reported that they had used the internet in the past 12 months to seek information that they needed.

Despite the near-ubiquitous use of the internet in Hong Kong households, online drug information searching is not commonly performed. Of the 443 respondents who participated in a local survey, less than half (44%) reported that they had looked for health information online, mostly using professional websites including government and hospital sites [11]. The majority of respondents surfed for disease-specific information and general information on healthy lifestyle. However, only 10.9% of respondents got online information on drugs and medications [11]. Respondents who were older, had lower than tertiary education, and had lower monthly income were less likely to surf online health information. Respondents also indicated that they were uncertain whether the information found online was reliable and accurate. Although this local study is limited by a convenience sampling approach, its finding suggests that there are indeed barriers hindering the implementation of ePI systems but research in this area is limited, and no other study has been performed to examine the specific situation in Hong Kong.

In Hong Kong, pharmacists play an important role in traditional drug distribution and dispensing duties. Over the recent years, a paradigm shift from mere product dispensing to more patient-oriented delivery of pharmacy service has been promoted [12]. Pharmacists are becoming more actively involved in making clinical decisions and patient care in both hospital and community settings. Hong Kong is also a hub for many international pharmaceutical companies. Most local country offices are focused on medical affairs and sales and marketing of pharmaceutical products in Asia. Other than practicing as authorized persons in this capacity, industrial pharmacists facilitate quality control, regulation, and registration of new products [12]. The execution of such services requires pharmacists of different practicing sectors to apply their professional drug information retrieval skills from multiple sources, particularly from package inserts. Consequently, obtaining product information from centralized ePI systems may be more efficient than using paper PI. Moreover, ePI may be used as a supporting platform to allow pharmacists to conduct patient education activities. A locally adapted set of ePI may improve information sharing and communication between patients and pharmacists, thereby empowering patients in the management of their health.

The objectives of this study were to explore working pharmacists’ overall perception of ePI, with a focus on gathering the perceived benefits and barriers that pharmacists face when they use ePI in their practice, and identifying potential challenges to the implementation of an ePI system in Hong Kong. It is expected that the findings of this study will reflect the acceptance level of pharmacists toward ePI and will yield insights into necessary improvements and objectives for future studies related to ePI.

## Methods

### Study Design

This was a mixed-method study that involved a structured questionnaire and individual interviews with practicing pharmacists in Hong Kong between January and May 2020. Approval from the Survey and Behavioral Ethics Committee of the Chinese University of Hong Kong was obtained before the initiation of this study (Reference number: SBRE-19-204). Written informed consent was obtained from all of the participants.

### Study Population

Pharmacists were eligible if they were (1) licensed to practice in Hong Kong, (2) self-identified as capable of reading and writing in English (as most drug information platforms available in Hong Kong are in English), and (3) currently working locally in any pharmacy-related sectors and institutions, including but not limited to public and private hospitals, community pharmacies, pharmaceutical companies, distributors, government authorities, pharmacy-related nongovernmental organizations, and private clinics. Eligible pharmacists were identified from the above sectors and recruited purposively using a snowball sampling approach. As the survey and interview were both conducted in Chinese, respondents must be able to converse in Cantonese and read Traditional Chinese. Eligible pharmacists were first approached via email where the study objectives were briefly described, and upon informal consent, they were subsequently contacted by phone to arrange for a face-to-face interview.

Research that has evaluated sample-size requirements in qualitative studies shows that data saturation often occurs at approximately the 12th to 16th respondent in a homogenous group [13,14]. Therefore, the minimum target sample size in this study was 12 respondents. After the 12th participant, recruitment was to continue until data saturation was reached.

### Study Procedures

#### Survey

After obtaining informed consent, the overarching aim of this research (ie, exploring the prospect of developing an ePI system in Hong Kong) was briefly described to pharmacists (respondents). They first completed a structured online questionnaire that was developed based on reported experience with ePI by other countries and regions [1-6], as well as consensus from research team comprising practicing pharmacists (LT, LQ, TC, and DC), senior pharmacy students (EF and GA-Y) and research methodologists (TL and YC). Functionality of the electronic survey was pilot tested on 2 research assistants. This was a closed survey where only respondents had access to the survey via a protected link. The survey consisted of 2 major sections.

The first section was a set of closed-ended questions to collect baseline characteristics, which included practicing sector and site and years of working experience. The second section consisted of questions to evaluate respondents’ level of agreement with each statement about the potential advantages of ePI over paper PI in different aspects, such as professionalism, usability, presentation, and environment. Some examples of the statements were “Using ePI instead of PI can enhance communication with patients” and “Using ePI instead of PI can enhance adequacy of drug information” (Multimedia Appendix 1). The agreement score was presented in a Likert scale ranging from 1 (ePI is much worse than paper PI) to 10 (ePI is much better than paper PI). The questionnaire was developed and administered in Traditional Chinese, an official written language in Hong Kong. It was translated to English for reporting purposes (Multimedia Appendix 1).

#### Structured Interview

After completing the online questionnaire, respondents were interviewed individually. The interviewers (EF and GA-Y) did not have prior relationship or interaction with the respondents. Their experience with using ePI and their perceptions of the benefits and challenges of citywide ePI implementation in Hong Kong were discussed systematically using a structured script (Multimedia Appendix 2). Their opinions on the method of implementation and the feasibility of a complete replacement of paper PI by ePI were also collected. All of the interviews were conducted in Cantonese, an official spoken language in Hong Kong, and were audio recorded. Each interview lasted between 20 and 40 minutes, and was held privately at the workplace of the respondents.

### Special Considerations During the COVID-19 Pandemic

Three interviews were completed in January 2020 during the pre-COVID-19 phase. The remaining 13 interviews were conducted between mid-April and May when the COVID-19 situation in Hong Kong had become relatively under control. Respondents were given a choice of whether they would like to have face-to-face or phone interviews, which all 13 respondents indicated that they preferred meeting in-person. The Prevention and Control of Disease (Prohibition on Group Gathering) Regulation (Cap. 599) came into operation on March 29, 2020 to prohibit any group gathering of more than 4 persons in any public place in Hong Kong [15]. Hence, each respondent completed the interview with only 2 investigators (EF and GA-Y). Basic social distancing measures (keeping a distance of at least 1 m from each other and wearing of facemasks) were strictly adhered to. In view of the ongoing pandemic, no repeat interview was conducted.

### Data Analysis

Online survey responses were recorded anonymously and retrieved electronically from the online survey software Qualtrics (SAP). Only completed survey was analyzed. All of the descriptive data collected from the survey were analyzed using the Statistics Package for Social Science, version 25 (IBM Corp.). The CHERRIES Checklist [16] was adopted to report the online survey findings.

All of the interviews were transcribed verbatim in Chinese. Data were managed using Archiv für Technik, Lebenswelt und Alltagssprache (ATLAS).ti version 8 qualitative data-analysis software (Scientific Software Development GmbH). Thematic analysis was adopted to analyze the qualitative data. Grounded theory was used to generate relevant themes and identify specific outcomes. Grounded theory was chosen as the theoretical framework as it is one of the most widely used systematic approaches to analyze qualitative data in the field of social sciences and health care [17,18]. There are limited data in the literature on pharmacists’ perception of ePI. The inductive nature of grounded theory is applied to obtain a deeper understanding of the rationale behind nascent barriers or perceived benefits of ePI that are unique to the local population.

Two researchers (EF and GA-Y) read all of the transcripts and coded them independently. Coding was conducted in 2 cycles: in the first cycle, codes were created and data segments were assigned to the codes; in the second cycle, the code lists were validated and then applied to the remaining data. Codes were then cross-checked and reviewed by a third researcher (YC). Thereafter, the coding and themes were discussed with the research team, and a coding framework was developed and applied to all of the transcripts. The number of recurring themes was noted accordingly. Finally, the themes and codes were translated into English for reporting purposes. The COREQ (COnsolidated criteria for REporting Qualitative research) 32-item checklist [19] was adopted to report the qualitative findings (Multimedia Appendix 3).

## Results

### Working Experience and Characteristics of Respondents

A total of 16 pharmacists were recruited, comprising 4 community pharmacists, 5 hospital pharmacists, and 7 industrial pharmacists (Table 1; response rate: 100%). The majority of pharmacists (respondents) reported having 1-10 years of working experience.

**Figure 1:**
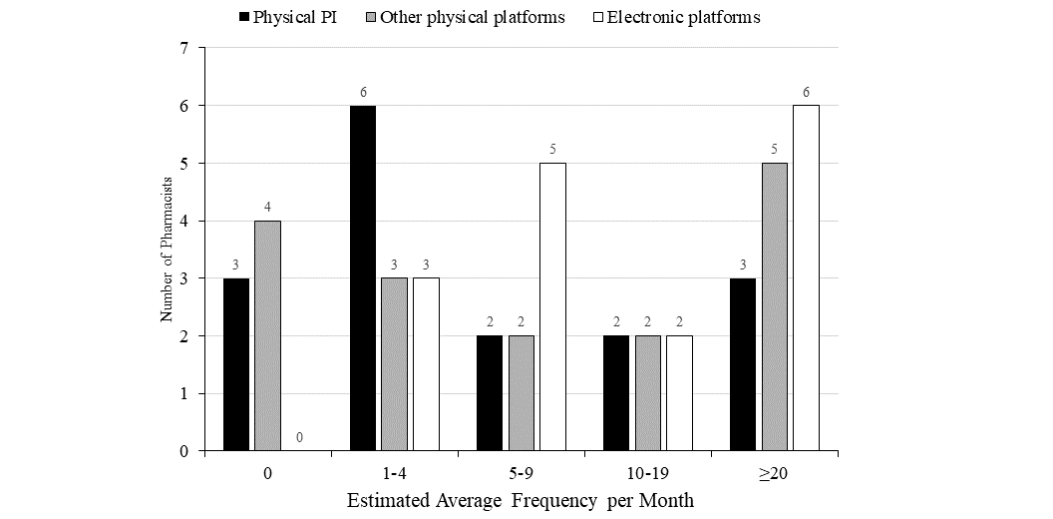
Frequency of retrieving drug information from different platforms (N=16). Physical PI refers to the conventional paper product information within the package of the drug product. Other physical platforms include paper copies of MIMS, BNF, LexiComp Handbook etc. Electronic platforms include electronic product information systems (eg, eMC UK, DailyMed/FDA), drug databases (eg, Lexicomp, Micromedex, AccessPharmacy), point-of-care databases (eg, ClinicalKey, UpToDate, Dynamed Plus), and Pharmaceutical (prescribing) references (eg, electronic MIMS, BNF). BNF: British National Formulary; eMC: Electronic Medicines Compendium; FDA: Food and Drug Administration; MIMS: Monthly Index of Medical Specialities; PI: product insert.

Almost all of the respondents considered themselves as regular retrievers of drug information (n=15/16, 94%), and all used electronic platforms at least once per month on average (n=16/16, 100%; Figure 1).

**Figure 2:**
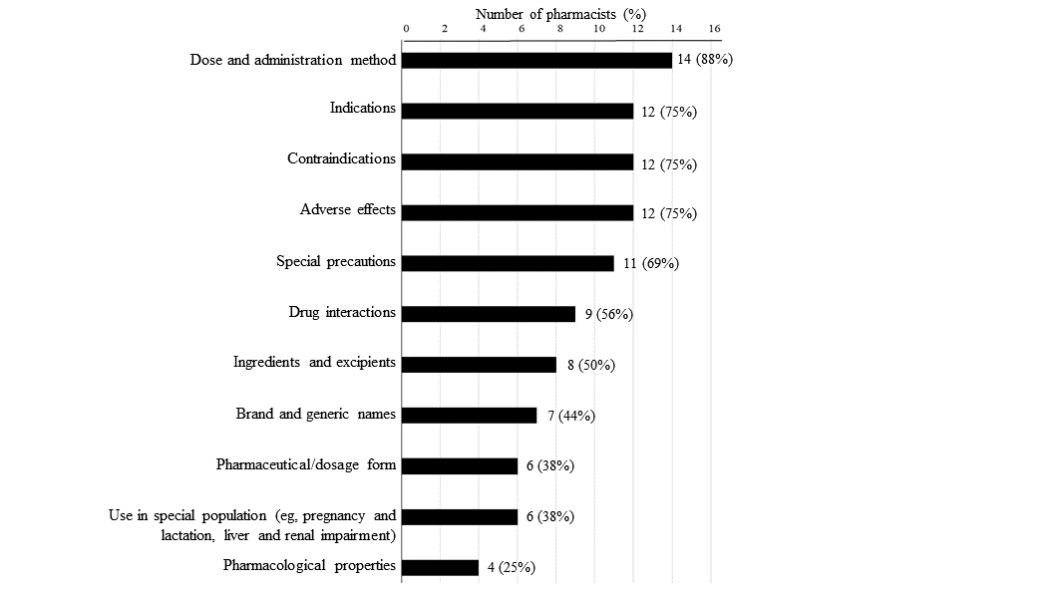
Categories of Drug Information Commonly Retrieved by Pharmacists (N = 16).

More than 85% (n=14/16, 88%) of the respondents commonly used package inserts or electronic platforms for retrieving information about dosages and methods of administration, and 75% (n=12/16) commonly retrieved information about indications, contraindications, and adverse effects of drugs (Figure 2).

**Table 1:** Characteristics of study population (N=16).

Characteristics			Number of respondents (%)
**Current practicing sectors**			
	Hospital	5 (31)	
	Community pharmacy	4 (25)	
	Multinational pharmaceutical company	7 (44)	
**Previous practicing sectors^a^**			
	Public hospital	8 (50)	
	Private hospital	2 (13)	
	Community pharmacy (chain or independent)	5 (31)	
	Multinational pharmaceutical company	7 (44)	
	Nongovernmental organizations	2 (13)	
	Others	3 (19)	
**Years of working experience**			
	1**-**5	5 (31)	
	5**-**10	3 (19)	
	10**-**15	1 (6)	
	15**-**20	3 (19)	
	Did not mention	4 (25)	
**Most frequently used electronic devices for accessing online drug information**			
	Laptop	6 (38)	
	Smartphone	10 (63)	

^a^Proportions do not add up to 100% as a pharmacist can have experience in multiple sectors.

### Survey

In general, respondents indicated that their experience with ePI had been entirely positive (Table 2). The speed with which drug information can be retrieved and the degree to which the drug information is up to date were considered the greatest strengths of ePI, being given an average score of 9.29 out of 10 by the respondents. The clarity with which ePI presents drug information to patients was considered as the weakest aspect of ePI, with an average score of only 6.93 out of 10.

**Table 2:** Pharmacists’ perceived impact of electronic product information (ePI) system on different aspects of their work.

Impacts of ePI		Mean score^a^ (SD)
**Professionalism**		
	Patient safety	7.14 (1.79)
	Communication with patients	6.93 (2.06)
	Communication with prescribers	8.07 (1.33)
**Usability**		
	Adequacy of drug information	7.93 (1.73)
	Retrieving speed	9.29 (0.73)
	Accessibility of drug information	8.64 (1.34)
	Degree to which the drug information is up to date	9.29 (0.83)
**Presentation**		
	Organization and layout	7.57 (1.74)
**Environment**		
	Impact on the environment	9.14 (0.77)
**Overall**		
	Overall impact	8.00 (1.30)

^a^The agreement score range was from 1 (ePI is much worse than paper PI) to 10 (ePI is much better than paper PI).

### Structured Interview

Data saturation was reached and no new coding was observed after 15 interviews. Five main themes were observed in the result, namely, the problem of paper package inserts and current electronic drug information sources (Theme 1), the advantages of ePI (Theme 2), the challenges of ePI implementation (Theme 3), concerns about the replacement of paper PI with ePI (Theme 4), and suggestions on how to implement a local ePI system in Hong Kong (Theme 5).

#### Theme 1: Problems With Paper Package Inserts and Current Electronic Drug Information Sources

Most respondents agreed that there are many obvious problems with paper package inserts, especially in their layout. In particular, it was noted that the font size on package inserts is too small, making the insert difficult to read:

The layouts of package inserts designed by different companies can be very different, and the fonts used are so small that I always have to spend a long time finding the information I want.

Respondent 01, industrial pharmacist

Another respondent stated an example:

When doctors call and ask questions, we usually have to answer within a few minutes, and it is too time-consuming to look for answers on package inserts.

Respondent 02, industrial pharmacist

Furthermore, 2 respondents stated that sometimes they cannot retrieve information from a package insert if they do not have the drug on hand or if the drug is in a sealed package. That is, respondents are not able to retrieve information from package inserts at their own convenience. In this situation, respondents indicated that they look for information on other electronic sources, via a search engine or electronic databases. The most frequently mentioned databases were the Monthly Index of Medical Specialities (MIMS) (n=9/16, 56%), Lexicomp (n=7/16, 44%), and Micromedex (n=6/16, 38%). However, some respondents stated that the information on MIMS is too general and that they cannot find the information they want. Another respondent pointed out there may be out-of-date or even wrong information on the MIMS website or in search engine results:

There is just general information on MIMS, such as storage conditions. There is not enough detailed information, such as clinical trial data. Some people may prefer a complete PI over MIMS.

Respondent 03, industrial pharmacist

#### Theme 2: Perceived Advantages of ePI

Overall, respondents noted that ePI had either user-related or technical advantages (Table 3). The most noted benefits of ePI were improved access to the most updated information and the reduced cost of printing paper leaflets.

**Table 3:** Major advantages and disadvantages of electronic product information identified by respondents.a

Advantages		Disadvantages	
**User related**			
	Facilitate retrieval of the most updated product labeling information		Need for patients to adapt to assess information electronically
	Improved patient access to product information		Patients’ preference for paper leaflet within the drug package instead of browsing a website for drug information
	Open access to information		Accessibility of information is limited as it is technology dependent
	Allow prompt communication of new safety information to health care professional/public		Not easy to highlight important points
	Facilitate alignment of information among different brands of the same drug		
**Technical related**			
	Reduce printing cost/redressing cost of package leaflet		Retrieval of information is not possible when there is no internet access
	Allow prompt implementation of new indication		Risk of security issues or cyber attack
	More environmentally friendly		Need for tight control and annotations over the different versions of electronic product information
		High cost for development and maintenance of a reliable platform	

^a^Interview transcripts were reviewed. Major themes on advantages and disadvantages of electronic product information were identified using thematic analysis.

Respondents considered the ease of retrieving drug information as the biggest advantage of ePI, as it enables them to do so without having the physical package inserts on hand.

The keyword searching function makes it more convenient to retrieve the information we want.

Respondent 04, industrial pharmacist

Respondents who worked in public hospitals stated that each hospital has a standard practice of scanning package inserts and uploading them to a centralized database to facilitate retrieval of drug information by doctors and pharmacists. They postulated that it would be more convenient if a formal ePI system were established. Another advantage identified was that ePI facilitates the retrieval of up-to-date information. For example, amendments or new information on the drug product can be annotated, updated, and disseminated electronically immediately after approval from regulatory authorities; this is a more timely and efficient approach, as compared to reprinting, repackaging, and distributing the revised paper PI. The update of new information can also be communicated to the user through a notification system or “pop up message.”

A key advantage of ePI for industrial pharmacists is being able to view the updated version of a package insert directly on a website, without any other procedures being required. For hospital pharmacists and community pharmacists, a key advantage of ePI is being able to ensure that the information they are viewing is up to date. Finally, 1 respondent pointed out that implementing an ePI system is more environmentally friendly than printing paper-package inserts.

#### Theme 3: Challenges of ePI Implementation

Despite the many advantages of ePI mentioned during the interviews, many respondents highlighted concerns about the security risks and high cost associated with system maintenance and that certain subpopulations may not be sufficiently computer literate to navigate the ePI system (Table 3).

Respondents mentioned that their biggest concern with the implementation of a local ePI system is that there may be a legal problem; specifically, that placing package inserts on a website may violate Cap.231 *Undesirable Medical Advertisements Ordinance* (UMAO), which states that no person shall publish, or cause to be published, any advertisement likely to lead to the use of any medicine for the purpose of treating human beings for any disease and conditions specified [10]. Examples of such conditions include gynecological or obstetrical disease, correction of deformity or the surgical alteration of a person’s appearance, and treatment of benign or malignant tumor. Thus, it will depend on the manner in which drug information is placed online. For example, a paper PI that is inside any container or package containing an oral drug does not constitute “publication of advertisement.” However, if a Quick Response (QR) code was printed on the outside of a package such that consumers could access the information by scanning the QR code, some respondents suggested that this may potentially violate the law. One respondent suggested that

It is illegal for a pharmaceutical company to publish information that claims a product has curative or preventive effects on cancer to be open to the public. Such locally published information may violate UMAO

Respondent 05, industrial pharmacist

Another challenge of implementation that was identified in interviews is that there is a low incentive for implementation of an ePI system in Hong Kong. One respondent explained that given the fact that drug information can be obtained from various existing sources, he does not expect governing authorities to take the initiative in developing a local ePI system. As compared to establishing an online drug information system, the government might allocate its resources to more pressing and ongoing “e-health projects,” such as setting up the electronic health record sharing system between private and public sectors, and the Chinese Medicine information system. Respondents were also skeptical on whether the pharmaceutical industry in Hong Kong is supportive of this initiative, though the 5 industrial pharmacists interviewed in this study (representing different pharmaceutical companies) seemed to agree with the rationale and anticipated benefits behind establishing an ePI system. In addition, the public’s potential level of acceptance of an ePI system is unknown, and local data on support for ePI initiatives are lacking.

Other difficulties in implementation were suggested to be the initiation and maintenance of the system. Some respondents thought that it will be difficult to include all drugs in a single system, as there are many registered pharmaceutical products in Hong Kong.

There are many registered brands for certain drugs in Hong Kong. Some community pharmacies may sell different brands of the same drug, and we notice that the package inserts of different brands can be slightly different.

Respondent 06, community pharmacist

Therefore, this respondent thought that the large number of registered pharmaceutical products from different companies may increase the difficulty of managing the labeling content of all drugs in an ePI system. For example, the potential challenges in the standardization of the ePI format were also mentioned, although standardization would enable faster retrieval of information. There is a need to establish systems and processes to enforce compliance with labeling and formatting standards; this may likely be cost intensive. Therefore, many respondents were concerned about the maintenance of the ePI system; 1 respondent pointed out that more resources may be needed for system maintenance:

If the information is updated more frequently, the Drug Office [the law enforcement agency that governs all legislations concerning medicines in Hong Kong] will have to review more changes of particulars, and more human resources may be needed.

Respondent 07, hospital pharmacist

The costs associated with implementation and system maintenance are issues of concern. For example, one respondent mentioned that the transition from paper PI to ePI itself might pose a major logistic burden to some pharmaceutical companies:

We have to convert the existing PI into ePI format. This is a significant challenge for the industry. We will probably need high-end IT support. If a company, like mine, has many registered products, this will require significant resources. For the smaller companies, they would still need to invest resources on ePI compliant systems.

Respondent 10, industrial pharmacist

#### Theme 4: Concerns With Complete Replacement of Paper PI With ePI

Some respondents were concerned about the accessibility of information, especially for the elderly population and patients with low computer literacy:

As we can see in hospitals, most of the patients are elderly, and they may not know how to access the information from the Internet.

Respondent 08, hospital pharmacist

Another respondent also shared a similar thought:

Many elderly people do not know how to use electronic devices, and they may rush to a community pharmacy and ask for help to retrieve online drug information, increasing the burden on us as community pharmacists.

Respondent 06, community pharmacist

The same respondent also expressed concern over what might happen in a situation of system breakdown:

If we rely totally on an online ePI system, in the situation of a system breakdown we will not have any other alternative ways to retrieve drug information.

Respondent 06, community pharmacist

Overall, most respondents suggested that an ePI system should be implemented and that the physical package insert system should also be maintained.

#### Theme 5: Enablers for Implementing a Citywide ePI System in Hong Kong

The majority agreed that a local ePI system should be initiated and managed by regulatory sectors in Hong Kong. A few respondents suggested that a centralized platform should be made for pharmaceutical companies to upload the latest version of their products’ ePI, and that the authority should take responsibility for verifying the data and ensuring their accuracy before releasing the information to the public.

The authority should set up some guidelines for the requirements of ePI format and information, so that pharmaceutical companies can know what to include and what to exclude in the drug information they upload onto an ePI system.

Respondent 09, industrial pharmacist

Most respondents also suggested that there should be separate information for both health care professionals and the public within the same ePI system. For health care professionals, detailed prescribing information and product-specific information should be provided. For the public, abridged patient-friendly information and nontechnical language should be used. Some respondents also suggested that patient-information leaflets in drug packaging inserts should be translated into Chinese, as they thought that a simplified patient-information leaflet in Chinese language would attract more patients to read the drug information:

The public will not be interested to read a long and complicated package insert, so a simpler version should be tailor-made for the public.

Respondent 10, industrial pharmacist

Finally, several features that can be integrated into an ePI system were mentioned, such as adding images of the pill and package. One respondent also suggested adding audio and video versions of drug information. Another suggested adding a *drug interactions* check function and *drug compatibility* check function:

Compatibility of a drug with other IV fluids is a common question, but sometimes information about compatibility is not included in the package insert, so it should be added to ePI system if possible.

Respondent 11, hospital pharmacist

## Discussion

### Principal Findings

In this study, we sought to determine pharmacists’ (our respondents’) perceptions of the benefits and challenges of implementing an ePI system in Hong Kong. Our respondents showed that pharmacists generally have a positive attitude toward ePI. Specifically, they stated that although they always use package inserts to retrieve drug information, they found many flaws in physical package inserts that can potentially be mitigated using ePI. However, they had mixed views on the replacement of paper PI by ePI; the majority thought that paper PI should be kept, rather than replacing with ePI. Respondents also voiced many concerns about the implementation and maintenance of a local ePI system. Most stated that further discussion and coordination of different stakeholders are needed before initiating a citywide ePI system in Hong Kong.

Our study shows that the majority of respondents reported frequent use of electronic databases to retrieve drug information, such as MIMS, Lexicomp, and Micromedex. However, several problems related to their use were identified. Currently, no study has systematically evaluated local pharmacists’ perception of existing online drug databases. However, anecdotal finding from our respondents, as well as reports from other countries, suggested that the common problems identified by health care professionals were the lack of comprehensive coverage of drug items, incomplete information (eg, missing information on drug compatibility for intravenous administration), and underutilization of internet features that enhance readability and understanding of medication information (eg, font enlargement, glossary of terms) [20,21]. As identified from a local study that reviewed local drug information use, authors found that despite the widespread use of the internet in Hong Kong, the public is still not benefiting from existing online drug information databases [22]. Authors identified that this was attributable to the readability of existing online drug information, as well as a language barrier, as most of the information is provided in English rather than Chinese [22]. The implementation of an ePI system is expected to ameliorate these problems, as it will offer a platform with comprehensive drug information in a standardized format for health care professionals, as well as patient-friendly information in Chinese for the public.

Several advantages of ePI were identified in this study. The ease of retrieving drug information is considered to be greatly enhanced by ePI, as users can access drug information whenever they need it, even when they do not have the drug package on-hand. The experience of other countries with ePI confirms that increased availability of drug information can be enabled by an ePI system [2,6,7]. In addition, online drug information can be constantly updated, which will assist the work of health care professionals in reducing drug-related problems in a hectic clinical setting. This is especially relevant to the context of Hong Kong health care system, where staff shortages, workforce problems, and timeliness of services are key shortcomings [12,23]. It is expected that a mature ePI system would serve as a one-stop drug information hub to help improve delivery of information to users.

Despite its many advantages, one of the challenges identified in the interviews with respondents was that an ePI may violate Cap.231 *UMAO*. According to Cap.231, any notice, poster, circular, label, wrapper, or document is considered to be an advertisement. The law states that no person shall publish, or cause to be published, any advertisement likely to lead to the use of any medicine for the purpose of treating human beings for any disease and conditions specified [24]. It is important to note that this law is targeted at advertisements that are published locally within the jurisdiction of Hong Kong. An ePI is technically not an “advertisement” by nature. The responders raised this point probably because they held a strict interpretation of the ordinance that ePI published online locally might be deemed as an “advertisement” under UMAO, and therefore a contravention of the ordinance. We also identified 2 exemptions concerning this ordinance. First, there are exemptions for providing health care professionals with drug information; the law also states that “it shall be a defense to prove that the advertisement to which the proceedings relate was made only in a publication of a technical character intended for circulation” mainly among medical practitioners, pharmacists, and the medical and para-medical staff of hospitals, clinics, and maternity homes [10]. This implies that the UMAO will not be violated if ePI system access is limited to health care professionals. Second, if open access to the public is desired, discussion with the government will be required. An amendment of the law may be necessary to exempt product information that is publicly available on the local ePI system from being referred as “advertisement.” Resources will also be required to manage the large database of drug information and to verify the information provided by manufacturers.

Although the respondents showed support for the implementation of a local ePI system, the majority wanted paper PI to be kept to serve as an alternative for special circumstances, such as during a system breakdown or other technical problems. In particular, respondents expressed concerns over the ability of the elderly population and patients with low computer literacy to use ePI. According to a study about online health information-seeking behavior in Hong Kong, a “digital divide” may exist, such that senior citizens and individuals of a lower socioeconomic status and a lower education level may be less likely to have access to online drug information [11]. This would be a problem, as Hong Kong has a growing elderly population who may have limited access to digital information. Accordingly, we propose that local champions should be identified to serve as advocates to help deliver and promote the benefits of ePI. Our findings also reveal the need for ePI-related education to be incorporated, starting from the undergraduate level to enable professional development and empower practitioners to effectively utilize ePI for drug-information retrieval.

In summarizing all recommendations raised by respondents, we have identified 2 viable approaches to establishing a centralized ePI system. The first approach is to take advantage of the current databases and drug websites that are managed by the local authorities, onto which the Drug Office can undergo transition into an XML format of PI, and followed by pharmaceutical companies during registration or during changes of the particulars of pharmaceutical products. Providing drug information in XML format should become a mandatory requirement after the successful transition. The second approach is to develop a new centralized platform where pharmaceutical companies submit drug information and labeling content of their pharmaceutical products according to predefined standards in XML format. Aside from being the developer and regulator of the ePI system, the government could also provide financial support for pharmaceutical companies to transit into the ePI system. It is also possible that pharmaceutical companies can collaborate and develop a consortium or consensus on the key principles, common standards, and governance process of the ePI system. The Hong Kong Association of the Pharmaceutical Industry may facilitate such initiative, together with other stakeholders including consumers, health care professionals, academic institutions, and regulatory authorities.

The collective opinions and recommendation gathered from the respondents suggest that an integrated drug information system should be implemented to serve both professional users and the public. Therefore, we propose that Hong Kong could adopt a similar platform as the Electronic Medicines Compendium (eMC), which is a well-developed ePI system used in the UK. eMC is managed by a third-party provider, and pharmaceutical companies pay to upload their SmPC and a package leaflet onto the eMC website [1]. There is a standardized format and layout for drug information on the website, with in-depth information on product characteristics supplied for health care professionals, and an abridged version available for patients and the public. The UK authorities also play a pivotal role in the management of this ePI system, as all drug information uploaded on the website must be checked and approved by the UK/European medicines licensing authority (Medicines and Healthcare products Regulatory Agency [MHRA]/EMA). Currently, the eMC has more than 10,600 documents that have been verified and approved by the UK or European government agencies that license medicines. This system is well-received by the citizens of the UK and EU, as well as other parts of the world. Hong Kong could benefit from adopting key features of the eMC system, as well as integration of other reputable and complementary sources of non-drug–related health information. For example, the system can include portal links to the public health care system website (Hospital Authority [25]), government websites (Hong Kong Department of Health [26], Centre for Health Protection [27], Chinese Medicine Regulatory Office [28]), or clinical trial portals and databases on adverse drug reactions. It is anticipated that this ePI system can be a leading, authoritative platform for health and drug information on the internet in Hong Kong.

### Limitations of Study

The study population comprised pharmacists only, who are presumably the health care professionals that most clinicians and the public would consult for drug-related inquiries. More studies are required to understand the acceptance levels of other health care professionals for ePI, including doctors and nurses, as they are also frequent users of package inserts. In addition, the sample size in this study was small. However, saturation was identified and no new coding was observed after 15 interviews. We also acknowledge that the data generated in the interviews broadly revolve around the needs of pharmacists, patients, and policy makers (eg, the government), with minimal references to other health care professionals. This might be due to the nature of the questions asked in the structured interviews, as there was not sufficient contact time to ask respondents to discuss other specific issues related to ePI. Given the nascent status of the ePI field in Hong Kong, we reason that despite these limitations, there is still reasonable credibility of these preliminary qualitative findings in demonstrating potential relevance of an ePI system in Hong Kong.

Study participants were recruited using purposive snowball sampling, and this may have resulted in selection bias. Although the respondents reported experience in multiple sectors of practices, only pharmacists who were currently practicing in hospital, community, and industrial settings participated in the study. Notably, these are also the 3 major sectors of practice for 80% of the pharmacists in Hong Kong [29]. We speculate that the identified benefits and challenges are generally reflective of the current opinions of local pharmacists. Lastly, there are methodological limitations associated with grounded theory approach. For example, the investigators conducted an extensive literature review on ePI before the initiation of this study. This might lead to the prejudgment or formation of predetermined constructs even before data analysis. To address this limitation, at least two investigators conducted coding independently to facilitate fine-tuning of the analysis and converge on a shared interpretation.

Given the aforementioned limitations of this study, we would like to emphasize that this study should be interpreted as an effort to generate future research directions in this field. We hope to take advantage of existing models in other countries and identify essential features of well-established and effective ePI systems. A follow-up study should then evaluate how these features can be adapted to serve the needs of health care professionals and patients in Hong Kong. Future studies should also seek the opinions and expertise of different stakeholders on ways to maintain an ePI system to ensure its sustainability. In particular, the attitudes of other health care professionals and patients toward electronic drug information should also be evaluated when new services are introduced and when they are more adapted to reading the electronic versions of patient drug-information leaflets.

### Conclusion

The establishment of a citywide system of ePI would be in keeping with the vision embodied by “The Smart City Blueprint for Hong Kong” [30], which was introduced by the Government of the Hong Kong SAR to facilitate the use of innovation and technology to improve people’s quality of living.

This qualitative study is the first step in exploring the feasibility of implementing a citywide ePI system in Hong Kong. Our findings suggest that pharmacists generally have a positive attitude toward ePI implementation, as they highlighted the advantages of ePI in enabling better updating and retrieval of drug information while also recognizing the many challenges that need to be overcome. In particular, Hong Kong pharmacists generally appear to not be in favor of a total replacement of paper PI by ePI. Nevertheless, the perceived benefits of ePI gathered from this study, as well as collective evidence from other countries with mature ePI systems, confirm that more efforts should be made to promote optimized development and implementation of an ePI system in Hong Kong.

## Appendix

Multimedia Appendix 1 Perceived Impact of Electronic Product Information (ePI) on Pharmacists’ Work (Questionnaire).

Multimedia Appendix 2 Structured Interviewer Guide.

Multimedia Appendix 3 CHERRIES Checklist.

## Abbreviations

ATLAS: Archiv für Technik, Lebenswelt und Alltagssprache
COREQ: COnsolidated criteria for REporting Qualitative research
EMA: European Medicines Agency
eMC: Electronic Medicines Compendium
ePI: electronic product information
EU: European Union
HTML: HyperText Markup Language
JSON: JavaScript Object Notation
MHRA: Medicines and Healthcare products Regulatory Agency
MIMS: Monthly Index of Medical Specialities
QR: Quick Response
SAR: Special Administrative Region
SmPCs: summary of product characteristics
UMAO: Undesirable Medical Advertisements Ordinance
XML: eXtensible Markup Language
