# Supplementary material for: Pharmacists’ Perceptions of the Benefits and Challenges of Electronic Product Information System Implementation in Hong Kong: Mixed-Method Study
Source: J Med Internet Res. 2020 Nov 10;22(11):e20765. doi: 10.2196/20765 (PMC7685919; doi:10.2196/20765)
Supplement: Multimedia Appendix 1 [file jmir_v22i11e20765_app1.docx]

電子化藥物包裝說明書例子 Example of an ePI system：

Electronic medicines compendium (EMC) (United Kingdom) https://www.medicines.org.uk/emc

從1-10分中，你認為使用電子化藥物包裝說明書比實體藥物包裝說明書會在以下範疇有什麼不同的影響?

What are the differences in the listed impacts when using ePI instead of conventional (paper) PI? Please choose on a scale from 1 (least impactful) to 10 (most impactful) that best indicates how you feel about ePI when compared to conventional (paper) PI.

**專業 Professionalism ***

1. 由實體藥物包裝說明書轉用電子化藥物包裝說明書有助提升病人安全

Using ePI instead of PI can enhance the level of patient safety

1. 由實體藥物包裝說明書轉用電子化藥物包裝說明書有助改善與病人的藥物資訊溝通
   Using ePI instead of PI can enhance communication with patients
2. 由實體藥物包裝說明書轉用電子化藥物包裝說明書有助改善與處方者的藥物資訊溝通

Using ePI instead of PI can enhance communication with prescribers

**可用性 Usability ***

1. 由實體藥物包裝說明書轉用電子化藥物包裝說明書有助提升藥物資訊的充足性
   Using ePI instead of PI can enhance adequacy of Drug Information
2. 由實體藥物包裝說明書轉用電子化藥物包裝說明書有助提升資訊檢索的速度
   Using ePI instead of PI can enhance speed with which product information can be retrieved
3. 由實體藥物包裝說明書轉用電子化藥物包裝說明書有助提升資訊檢索的可接觸度
   Using ePI instead of PI can enhance accessibility of product information
4. 由實體藥物包裝說明書轉用電子化藥物包裝說明書有助提升資訊更新的速度
   Using ePI instead of PI can enhance degree to which product information retrieved is up to date

**表達 Presentation ***

1. 由實體藥物包裝說明書轉用電子化藥物包裝說明書有助改善與藥物包裝說明書的組織及排版
   Using ePI instead of PI can enhance the organization and layout of which medication information is presented

**環境 Environment ***

1. 由實體藥物包裝說明書轉用電子化藥物包裝說明書有助減低對環境的影響
   Using ePI instead of PI can reduce the impact on the environment

**整體 Overall ***

1. 由實體藥物包裝說明書轉用電子化藥物包裝說明書有良好的整體影響

Using ePI instead of PI have a positive overall impact

*The electronic questionnaire consisted of five pages (one section per page). A “review” function was available at the end of the questionnaire before submission.

The questionnaire was developed and administered in Traditional Chinese, an official written language in Hong Kong. It was translated to English for reporting purposes. Two investigators (FWT and AYG, both effective bilingual) translated the questionnaire from Chinese to English independently; differences in terminologies were discussed and a third investigator (YTC) reviewed a final reconciled version.

We decided a priori that duplicate database entries having the same user ID or IP address would be eliminated before analysis. The most recent entry would be used. However, no participants submitted multiple entries.
