# Supplementary material for: Pharmacists’ Perceptions of the Benefits and Challenges of Electronic Product Information System Implementation in Hong Kong: Mixed-Method Study
Source: J Med Internet Res. 2020 Nov 10;22(11):e20765. doi: 10.2196/20765 (PMC7685919; doi:10.2196/20765)
Supplement: Multimedia Appendix 2 [file jmir_v22i11e20765_app2.docx]

Part I. Disclaimer and Personal Experience

1. 你過往或現時有否參與在香港設立電子化藥物包裝說明書系統的計劃？

Have you participated in or plan to establish an electronic product information (ePI) system in Hong Kong?

1. 你過往及現時在那個領域中工作過，工作了多久，有甚麼主要職責？

What are your main responsibilities in the current sector/site that you are practicing? How many years of experience do you have in this field?

Part II. Identifying Potential Challenges

1. 就在香港設立一個電子化藥物包裝說明書系統，你認為會有什麼困難？
   - - - 製藥業對此議題有沒有共識？
       - 運用統一電子格式 (XML) 的成本對部分持分者而言會否太高？

- 政策規則方面有甚麼限制 ？

What challenges do you foresee with developing an ePI system in Hong Kong?

- Does the pharmaceutical industry have a consensus on this issue?
- Will the cost of developing a centralized electronic format (XML) be too high for some stakeholders?
- What are the restrictions on policy rules?

1. 就在香港全面以電子化藥物包裝說明書取締傳統藥物包裝說明書，你認為會有什麼困難 ?

- 你接觸的病人當中有多少對電子科技不熟識或缺乏硬件、互聯網的病人？
- 你接觸的醫療專業人員有多大可能反對轉用電子包裝說明書系統？

What challenges do you foresee if conventional (paper) PI is completely replaced by ePI in Hong Kong?

- Are the patients you interact with familiar with using the Internet?
- Are the medical professional you are in contact with willing to switch to an ePI system?

All of the interviews were conducted in Cantonese, an official spoken language in Hong Kong. It was translated to English for reporting purposes. Two investigators (FWT and AYG, both effective bilingual) translated the questionnaire from Chinese to English independently; differences in terminologies were discussed and a final reconciled version was reviewed by a third investigator (YTC).
