# Supplementary material for: Pharmacists’ Perceptions of the Benefits and Challenges of Electronic Product Information System Implementation in Hong Kong: Mixed-Method Study
Source: J Med Internet Res. 2020 Nov 10;22(11):e20765. doi: 10.2196/20765 (PMC7685919; doi:10.2196/20765)
Supplement: Multimedia Appendix 3 [file jmir_v22i11e20765_app3.docx]

| Item Category | Checklist Item | Description | Page |
| --- | --- | --- | --- |
| Design |  |  |  |
|  | Describe survey design | Cross-sectional, snowball sampling | 9 |
| IRB (Institutional Review Board) approval and informed consent process | | |  |
|  | IRB approval | Approved by the Survey and Behavioral Ethics Committee of the Chinese University of Hong Kong was obtained before the initiation of this study (Reference number: SBRE-19-204). | 9 |
|  | Informed consent | Written informed consent was obtained from all participants. | 9 |
|  | Data protection | Participants were assured that their responses would be anonymous, though the researchers may still be able to identify the participants due to the small sample size, and the fact that it is a closed survey. Participants were also assured that only aggregated data the survey would be reported. | 12 |
| Development and pre-testing | | |  |
|  | Development and testing | The survey was developed based on reported experience with ePI by other countries and regions, as well as consensus from research team. Functionality of the survey was pilot-tested on two research assistants. | 10 |
| Recruitment process and description of the sample having access to the questionnaire | | | |
|  | Open survey versus closed survey | “Closed survey”. | 10 |
|  | Contact mode | There is no initial contact with the potential participants. | 11 |
|  | Advertising the survey | No advertisement as it is a closed survey. | NA |
| Survey administration | | |  |
|  | Web/E-mail | A password protected link is sent via email to the participants. | 10 |
|  | Context | Not applicable | NA |
|  | Mandatory/voluntary | Not applicable | NA |
|  | Incentives | No incentives. | NA |
|  | Time/Date | Survey was sent to the participants before the structured interview. | 10 |
|  | Randomization of items or questionnaires | No randomization of items. | NA |
|  | Adaptive questioning | No adaptive questioning was used. | NA |
|  | Number of Items | 1 section per page (total 5 pages) | Supple 1 |
|  | Number of screens (pages) | 1 section per page (total 5 pages) | Supple 1 |
|  | Completeness check | There was a function to check for completeness f the survey. Respondents would be directed back to the survey if they did not complete all 10 questions. Selection of one response option was enforced. | Supple 1 |
|  | Review step | Respondents were able to use a “review” button to display a summary of the responses before submission | Supple 1 |
| Response rates | | |  |
|  | Unique site visitor | Not applicable | NA |
|  | View rate (Ratio of unique survey visitors/unique site visitors) | Not applicable | NA |
|  | Participation rate (Ratio of unique visitors who agreed to participate/unique first survey page visitors) | Not applicable as it is a closed survey. All participants completed the survey. | NA |
|  | Completion rate (Ratio of users who finished the survey/users who agreed to participate) | Not applicable | NA |
| Preventing multiple entries from the same individual | | |  |
|  | Cookies used | We decided a priori that duplicate database entries having the same user ID or IP address were eliminated before analysis. The most recent entry would be used. However, no participants submitted multiple entries. | Supple 1 |
|  | IP check | IP address was used to identify potential duplicate entries from the same user. | Supple 1 |
|  | Log file analysis | Not applicable as no participants submitted multiple entries | NA |
|  | Registration | Participants need to login before completing the survey. Entries having the same user ID or IP address would be eliminated before analysis. The most recent entry would be used. However, no participants submitted multiple entries. | NA |
| Analysis | | |  |
|  | Handling of incomplete questionnaires | Only completed surveys were analyzed. | 12 |
|  | Questionnaires submitted with an atypical timestamp | Time required to complete the survey was not captured. | NA |
|  | Statistical correction | Not applicable due to the small sample size. | NA |

Eysenbach G. Improving the quality of web surveys: The Checklist for Reporting Results of Internet E-Surveys (CHERRIES). Journal of Medical Internet Research 2004; 6(3):e34. PMID: 15471760.

Supplement 4: COREQ 32-item checklist

|  | Item | Description | Page |
| --- | --- | --- | --- |
|  | **Domain 1: Research team and reflexivity** | |  |
|  | *Personal Characteristics* | |  |
| 1. | Interviewer/facilitator | Fung EWT and Au-Yeung GTF conducted the interviews. | 11 |
| 2. | Credentials | Fung EWT, B(Pharm)  Au-Yeung GTF, B(Pharm)  Tsoi LM, B(Pharm)  Qu L, B(Pharm), MCP  Cheng TKW, B(Pharm)  Chong DW, B(Pharm), MBA  Lam TTN, PharmD, PhD  Cheung YT, PhD | Title page |
| 3. | Occupation | Fung EWT – Final year (honors) student, Bachelor of Pharmacy program  Au-Yeung GTF – Final year (honors) student, Bachelor of Pharmacy program  Tsoi LM – Pharmacist, 3 years’ experience  Qu L – Pharmacist, 5 years’ experience  Cheng TKW – Pharmacist, 5 years’ experience  Chong DW – Senior Pharmacist, >10 years’ experience  Lam TTN – Lecturer and Senior Pharmacist, >10 years’ experience  Cheung YT– Epidemiologist and Assistant Professor | 10 |
| 4. | Gender | Fung EWT – female  Au-Yeung GTF – male  Tsoi LM – female  Qu L – female  Cheng TKW – male  Chong DW – male  Lam TTN – male  Cheung YT – female | NA |
| 5. | Experience and training | Fung EWT – Clinical pharmacy and biostatistics  Au-Yeung GTF – Clinical pharmacy and biostatistics  Tsoi LM – Clinical pharmacy  Qu L – Clinical pharmacy, regulatory affairs  Cheng TKW – Clinical pharmacy  Chong DW – Clinical pharmacy, regulatory affairs, business management  Lam TTN – Clinical pharmacy, biostatistics and research methodology (quantitative and qualitative)  Cheung YT – Clinical pharmacy, biostatistics and research methodology (quantitative and qualitative) | NA |
|  | *Relationship with participants* | |  |
| 6. | Relationship established | No relationship between the researchers and the participants was established before study commencement. However, the pharmacist community in Hong Kong is small; some participants and researchers do know each other on a professional level. | NA |
| 7. | Participant knowledge of the interviewer | Fung EWT and Au-yeung GTF (the main interviewers) are college students, so all participants, who are practicing pharmacists, did not have prior interaction with them before the interview. | 11 |
| 8. | Interviewer characteristics | The general aim of the study was briefly made known to the participants before the interview. | 10 |
|  | **Domain 2: study design** | |  |
|  | *Theoretical framework* | |  |
| 9. | Methodological orientation and Theory | Grounded theory was used. | 12 |
|  | *Participant selection* |  |  |
| 10. | Sampling | Snowball sampling was adopted. | 9 |
| 11. | Method of approach | Approached via email, then phone to confirm interview date/time and venue. | 10 |
| 12. | Sample size | 16 | 13 |
| 13. | Non-participation | All pharmacists whom we approached agreed to participate in the study. | 13 |
|  | *Setting* |  |  |
| 14. | Setting of data collection | Interviews were held at the workplace. | 11 |
| 15. | Presence of non-participants | Only the participants and interviewers were present. | 11 |
| 16. | Description of sample | Presented in Table 1. | 13, Table 1 |
|  | Data collection |  |  |
| 17. | Interview guide | Interviewers followed an interview guide. The questions were developed based on a literature review and consensus from the researchers. | Supp 2 |
| 18. | Repeat interviews | No repeat interviews. | 12 |
| 19. | Audio/visual recording | Audio recording was used. | 11 |
| 20. | Field notes | Field notes were made by the interviewers. | 11 |
| 21. | Duration | 20 to 40 minutes | 11 |
| 22. | Data saturation | The minimum target sample size in this study was 12 respondents. After the 12^th^ participant, recruitment was to continue until data saturation was reached. | 10 |
| 23. | Transcripts returned | Transcripts were not returned to participants. However, the report was shared with the participants upon request. | NA |
|  | **Domain 3: analysis and findings** | |  |
|  | *Data analysis* |  |  |
| 24. | Number of data coders | 2 independent data coders and 1 third coder to resolve discrepancies. | 12 |
| 25. | Description of the coding tree | Described under “Data analysis”. | 12 |
| 26. | Derivation of themes | Themes were derived from the data. | 12 |
| 27. | Software | (ATLAS).ti 8 | 12 |
| 28. | Participant checking | The report was shared with the participants upon request. | NA |
|  | *Reporting* |  |  |
| 29. | Quotations presented | Participant quotations were presented to illustrate the themes /findings. Participants were identified by a participant number and the practice sector. | Results section |
| 30. | Data and findings consistent | There is consistency between data and findings. We also identified consistency between the qualitative data and the survey results. | Results section |
| 31. | Clarity of major themes | The 5 major themes were presented as subsections (“Theme 1”, “Theme 2” etc.) in the narrative. | Results section |
| 32. | Clarity of minor themes | Minor themes were presented under the 5 major themes in the narrative. | Results section |

Developed from: Tong A, Sainsbury P, Craig J. Consolidated criteria for reporting qualitative research (COREQ): a 32-item checklist for interviews and focus groups. *International Journal for Quality in Health Care*. 2007. Volume 19, Number 6: pp. 349 – 357
